# Limited clinical relevance of mitochondrial DNA mutation and gene expression analyses in ovarian cancer

**DOI:** 10.1186/1471-2407-8-292

**Published:** 2008-10-08

**Authors:** Piotr Bragoszewski, Jolanta Kupryjanczyk, Ewa Bartnik, Andrea Rachinger, Jerzy Ostrowski

**Affiliations:** 1 Department of Gastroenterology and Hepatology, Medical Center for Postgraduate Education at the Maria Sklodowska-Curie Memorial Cancer Center and Institute of Oncology, Roentgena 5, Warsaw, Poland; 2 Department of Molecular Pathology, the Maria Sklodowska-Curie Memorial Cancer Center and Institute of Oncology, Roentgena 5, Warsaw, Poland; 3 Institute of Genetics and Biotechnology, University of Warsaw, Warsaw, Poland; 4 Institute of Biochemistry and Biophysics, Pawinskiego 5, Warsaw, Poland; 5 Marie-Curie Scholarship, Institute of Biochemistry and Biophysics, Pawinskiego 5, Warsaw, Poland

## Abstract

**Background:**

In recent years, numerous studies have investigated somatic mutations in mitochondrial DNA in various tumours. The observed high mutation rates might reflect mitochondrial deregulation; consequently, mutation analyses could be clinically relevant. The purpose of this study was to determine if mutations in the mitochondrial D-loop region and/or the level of mitochondrial gene expression could influence the clinical course of human ovarian carcinomas.

**Methods:**

We sequenced a 1320-base-pair DNA fragment of the mitochondrial genome (position 16,000-750) in 54 cancer samples and in 44 corresponding germline control samples. In addition, six transcripts (*MT-ATP6, MT-CO1, MT-CYB, MT-ND1*, *MT-ND6*, and *MT-RNR1*) were quantified in 62 cancer tissues by real-time RT-PCR.

**Results:**

Somatic mutations in the D-loop sequence were found in 57% of ovarian cancers. Univariate analysis showed no association between mitochondrial DNA mutation status or mitochondrial gene expression and any of the examined clinicopathologic parameters. A multivariate logistic regression model revealed that the expression of the mitochondrial gene *RNR1 *might be used as a predictor of tumour sensitivity to chemotherapy.

**Conclusion:**

In contrast to many previously published papers, our study indicates rather limited clinical relevance of mitochondrial molecular analyses in ovarian carcinomas. These discrepancies in the clinical utility of mitochondrial molecular tests in ovarian cancer require additional large, well-designed validation studies.

## Background

Mitochondria are most notably involved in ATP production but also contribute to thermogenesis, free radical production, calcium homeostasis, and apoptosis [1]. ATP production is required for most cellular functions, and cellular energy requirements are largely met by the mitochondrial oxidative phosphorylation system. Mammalian mitochondria contain ~1000 proteins [2] but only 13 of them are encoded by the mitochondrial genome [3]. Other mitochondrial proteins are encoded by nuclear DNA, synthesized in the cytoplasm, and imported by the mitochondria.

Although several mitochondrial proteins [4,5] form the nucleoid, which is a mitochondrial DNA-protein complex, their protective effects against mutagenesis are much less strong than those in the nuclear genome [6]. In addition, the high rate of mitochondrial DNA (mtDNA) mutation, which is several times higher than the rate for nuclear DNA [6-8], is likely explained by the production of reactive oxygen species by the mitochondrial oxidative phosphorylation system [8-10]. Increased accumulation of mtDNA somatic mutations has been reported in aging tissues such as brain, skeletal muscle, and fibroblasts [7,11,12] and in many pathological conditions including neurologic, metabolic, and age-related disorders. These alterations are especially prevalent in preneoplastic lesions and in human cancers, including breast cancer, ovarian cancer, colorectal cancer, gastric cancer, hepatic cancer, esophageal cancer, prostate cancer, and thyroid cancer [13-31]. Most studies on mtDNA mutation have focused on sequencing of the D-loop region.

Somatic mutations can occur throughout the mitochondrial genome sequence [14,17,26,28,32-34], but two hypervariable regions (HV1 and HV2) of the D-loop represent mutational hot spots [17,35]. The mononucleotide repeat between nt 303 and 309 is the most unstable microsatellite sequence within the non-coding D-loop region. This microsatellite sequence forms a persistent DNA-RNA hybrid that initiates replication of the mtDNA heavy strand [36]. Mutations within the C-stretch region from nt 303 to 309 may alter the regulation of mtDNA replication and, consequently, the mtDNA content in tumours. Whereas an increased number of mitochondria were found in thyroid cancer tissue [37], a decreased copy number of mtDNA was described in hepatocellular carcinoma harboring D-loop mutations [38].

The mitochondrial circular DNA is almost completely and symmetrically transcribed from its heavy and light strands, and the resulting polycistronic RNA is processed by endonucleolytic cleavage [39]. Mitochondrial gene expression parallels changes in the copy number of mitochondrial DNA [40,41]. The replication origin for the leading strand, the heavy- and light-strand promoters, their mitochondrial transcription factor A (mTFA) binding sites and the three conserved sequence blocks [3,42] are located within the displacement-loop (D-loop) of the mitochondrial genome. The mtTFA enhances mitochondrial DNA transcription in a promoter-specific fashion, in the presence of mitochondrial RNA polymerase and transcription factor B [43]. It seems that mtTFA is also crucial for replication [44] and for maintaining of mitochondrial DNA [5].

The D-loop is a control site for expression of the mitochondrial genome. Mutations which are located in regulatory elements of the D-loop region have been associated with the reduction in the ND6 expression and in the mtDNA copy number [42]. It seems therefore that functional location of some mutations within the control region may alter the rate of mtDNA transcription and replication.

Mitochondria are central executors of apoptosis, which is the main mechanism of tumor cell loss during chemotherapy. MtDNA may determine the cellular response to some anticancer agents [45-47], and mutations in the D-loop region have been correlated with resistance to FU-based chemotherapy in colon cancer patients [48]. As an independent prognostic factor in cancer patients, somatic mutation of mtDNA might also serve as a potential tumour marker [48].

Thus, many studies have proposed that mtDNA mutations might serve as both biomarkers of carcinogenesis and as predictive factors for the disease course. Our study analyzed the DNA sequence of the D-loop region and the expression of mitochondrial genes in ovarian cancer samples, and the association of molecular findings with clinical endpoints was evaluated. No significant relationship was found between mitochondrial alterations and clinical characteristics of the studied patients.

## Methods

### Patients

Ovarian cancer specimens were obtained from patients with FIGO IIC-IV disease during the initial surgical treatment prior to chemotherapy [49]. One to 10 tumour's fragments, depending on the tumour size, were snap frozen and stored at -72°C until use. Then, histological evaluation of the group of 106 tumours and over 300 of their tumour fragments was performed to control their relative content of non-tumour tissue. Several series of cryostat sections were prepared from different parts of each tumour's fragment. Upper and lower sections from each cryosection collection were evaluated by the pathologist (JK), and the rest of the tissue material was used in the study if it contained up to 5% fraction of stromal cells. Altogether, we selected 62 cancers which were represented by at least one cryosection collection containing ≥ 95% cancer cells. Forty-four matched peripheral blood samples served as germline controls. The study protocol was approved by the local Bioethical Committee, and all patients signed an informed consent form before inclusion.

The tumours were classified histologically (JK) according to the criteria of the World Health Organization [50] and were graded on a 4-grade scale according to the Broders' criteria [51]. There were 59 serous carcinomas and 3 cancers of other types; 5 tumours showed moderate differentiation (G2), 42 were poorly differentiated (G3), and 15 were mostly or completely undifferentiated (G4).

Twenty-seven patients were treated with platinum-cyclophosphamide therapy, and 35 patients were treated with taxane-platinum therapy. The criteria for evaluation of clinical endpoints were given previously [52]. In particular, platinum sensitivity was defined as complete remission, with disease-free survival ≥ 180 days. Other tumours were described as resistant to chemotherapy. The *TP53 *gene status of the tumours was analyzed previously [52].

### Sequencing of mtDNA D-loop

Standard molecular biology techniques were used. Total genomic DNA from tissue samples was purified with the DNeasy Tissue Kit (Qiagen GmbH, Hilden, Germany). The D-loop sequence of mtDNA was PCR amplified with primers D1F/D3R (Table 1). PCR products were sequenced in two directions by fluorescent dideoxysequencing on an ABI Prism 3100 Sequence Detection System (Applied Biosystems, Foster City, CA) with primers listed in Table 1.

**Table 1:** Sequences of oligonucleotide primers used in real-time PCR and sequencing reactions

**Primer pairs used in real-time PCR reactions**		
Gene	Primer sequence

*ACTB*	Forward: 5'-TGCGTTACACCCTTTCTTGACA
	Reverse: 5'-GCAAGGGACTTCCTGTAACAATG
*GAPDH*	Forward: 5'-GAAGGTGAAGGTCGGAGTC
	Reverse: 5'-GAAGATGGTGATGGGATTTC
*MT-ATP6*	Forward: 5'-TAGCCATACACAACACTAAAGGACGA
	Reverse: 5'-GGGCATTTTTAATCTTAGAGCGAAA
*MT-CO1*	Forward: 5'-GACGTAGACACACGAGCATATTTCA
	Reverse: 5'-AGGACATAGTGGAAGTGAGCTACAAC
*MT-CYB*	Forward: 5'-ATCACTCGAGACGTAAATTATGGCT
	Reverse: 5'-TGAACTAGGTCTGTCCCAATGTATG
*MT-ND1*	Forward: 5'-CCACCTCTAGCCTAGCCGTTTA
	Reverse: 5'-GGGTCATGATGGCAGGAGTAAT
*MT-ND6*	Forward: 5'-CAAACAATGTTCAACCAGTAACCACTAC
	Reverse: 5'-ATATACTACAGCGATGGCTATTGAGGA
*MT-RNR1*	Forward: 5'-TAGAGGAGCCTGTTCTGTAATCGAT
	Reverse: 5'-CGACCCTTAAGTTTCATAAGGGCTA
*UBC*	Forward: 5'-ATTTGGGTCGCGGTTCTTG
	Reverse: 5'-TGCCTTGACATTCTCGATGGT
	
**Primers used in sequencing reactions**	

Primer name	Primer sequence

D1F ^A^	Forward: 5'-AATGGGCCTGTCCTTGTAG
D2F	Forward: 5'-CGACATCTGGTTCCTACTTC
D3F	Forward: 5'-CGCTTCTGGCCACAGCAC
L16112F	Forward: 5'-CACCATGAATATTGTACGGT
D2R	Reverse: 5'-GGGTTTGGTTGGTTCGGG
D3R ^A^	Reverse: 5'-GGTGTGGCTAGGCTAAGC
DLP4R	Reverse: 5'-GTGGAAAGTGGCTGTGCAG
H16220R	Reverse: 5'-TTGATTGCTGTACTTGCTTGTAAG
H16540R	Reverse: 5'-GTGGGCTATTTAGGCTTTATGACCCTG

^A ^Primers used for PCR amplification of sequenced mtDNA fragment.

### Real-time RT-PCR analysis

Specific RNA concentrations were determined by real-time reverse transcriptase (RT)-PCR. Total tissue RNA was isolated with the RNeasy Mini Kit and QIAshredder columns (Qiagen GmbH, Hilden, Germany). Reverse transcription was performed with the SuperScript II Reverse Transcriptase (Invitrogen Co., Carlsbad, CA) reagent set according to the manufacturer's instructions. Quantitative evaluation of mRNA was performed on an ABI Prism 7000 Sequence Detection System with a 25-μl reaction mixture containing 12.5 μl 2× SYBR Green PCR Master Mix (Applied Biosystems, Foster City, CA), 5 μl cDNA, and 50 nM primers. Oligonucleotide primers for the analyzed gene transcripts were designed with Primer Express Software (Applied Biosystems, Foster City, CA) and are listed in Table 1. For each run, standard curves were generated for a primer set by serial dilution of pooled cDNA to counterbalance variations in PCR reaction efficiency. Melting curves were generated after each reaction to verify the melting temperature of the amplicon. In addition, the purity of the RT-PCR product was verified by agarose gel electrophoresis. To normalize nonspecific variations in real-time PCR, the normalization factor was calculated as the geometric mean of RNA concentrations of three control genes, *glyceraldehyde-3-phosphate dehydrogenase*, *ubiquitin C*, and *β-actin*.

### Statistical analysis

All continuous variable data were presented as median and range and were analyzed with the Mann-Whitney *U *test. The two-tailed Fisher's exact test was used to determine the relationships between categorical variables and D-loop mutations. Tumour response to chemotherapy was evaluated with the multivariate logistic regression model. Factors were selected with the backward selection technique, in which factors not significant at 0.1 were removed stepwise from the model. Initial multivariate models included the following variables: age of patient, FIGO stage, residual tumour size, grade, *TP53 *mutation, first-line therapy, haplogroup, D-loop somatic mutations, and expression of *RNR1*, *ATP6*, *CO1*, *CYB*, *ND1*, and *ND6*. Gene expression variables and age of patients were categorized by the median values.

*P *values of less than 0.05 were considered significant. Calculations were performed with the STATA 6.0 program (Stata Corporation 1999, College Station, TX).

## Results

### Variability of mtDNA in ovarian cancer

We sequenced a 1320-base-pair DNA fragment of the mt genome (positions 16000-750), which included the control region with the D-loop (positions 16024-576), in 54 cancer tissue samples and in 44 corresponding germline control samples. The complete sequencing results are available at . The obtained sequences were compared with the revised human mitochondrial Cambridge reference sequence deposited in GenBank (accession number AC_000021). Most of the sequence variations were identified as neutral polymorphisms of human mtDNA because they were found in both tumour and control DNA samples.

The D-loop sequences from the 54 ovarian cancer samples were used to assign patients to specific haplogroups, according to the method described by Finnilla et al. and Torroni et al. [53,54]: H (*n *= 27), U (*n *= 7), T (*n *= 6), J (*n *= 3), K (*n *= 3), unspecified (*n *= 3), I (*n *= 2), W (*n *= 2), and V (*n *= 1). The frequency of haplogroup variants found in the ovarian cancer patients was similar to that of the Polish population [55] (not shown), which supports the accuracy of the sequencing data obtained in this study [56]. Because most haplogroups were represented by only a few patients, to simplify further analyses, we divided patients into two haplogroups: H and all others. No significant association of tumour mutations with either of these two groups was found (Table 2).

**Table 2:** Results of statistical analyses of associations between D-loop mtDNA mutation and clinicopathologic parameters in 44 ovarian cancer patients

Parameter	Number of patients	Number of patients with mutated tumour	Number of patients with non-mutated tumour	*P*-value
Overall	44	25	19	

Age (median = 53)				
> 53	19	13	7	
≤ 53	24	12	12	0.3175

Grading				
G2 + G3	31	20	11	
G4	12	5	7	0.3014

FIGO stage				
II-IIIB	10	5	5	
IIIC, IV	34	20	14	0.7233

Residual tumour				
> 2 cm	14	9	5	
0–2 cm	29	16	14	0.534

*TP53 *mutation				
Yes	23	14	9	
No	20	11	10	0.7613

Response to chemotherapy				
Platinum-based				
Yes	10	5	5	
No	9	5	4	1
Taxane/platinum-based				
Yes	12	9	3	
No	12	6	7	0.2262
Both types of therapy				
Yes	22	14	8	
No	21	11	11	0.5434

Haplogroups				
H	22	14	9	
Others	21	11	10	0.7613

Statistical significance was analyzed with the two-tailed Fisher's exact test.

The sequencing results from 44 paired tumour and matching blood samples were used to search for differences that distinguished tumour mtDNA from blood mtDNA. Thirty-three somatic mutations were found in 25 of 44 (57%) ovarian cancer samples. The most frequent alterations were changes in length of homopolymeric C-tracts, which were found in 18 paired samples (positions 303–315, *n *= 15; positions 16184–16193, *n *= 3). The remaining 15 somatic mutations were substitutions detected in 11 specimens. Whereas the majority of length changes were heteroplasmic (15 of 18), most of the substitutions were homoplasmic (13 of 15).

Four of the substitutions, m.565A>G, m.586G>A, and heteroplasmic m.567A>C and m.16034G>A, were not recorded in the mtDBase database [57]. However, the m.586G>A mutation was found previously in colonic crypts, as recorded in the MITOMAP database [58]. Five other mutations were relatively rare: m.16193C>T (13/1854), m.16218C>T (9/1858), m.16390G>A (62/1805), m.16391G>A (17/1850), and m.16465C>T (4/1863). In parentheses are the numbers of records in the mtDBase database with/without this variant. Interestingly, in two cases the blood samples had an uncommon mtDNA sequence that was reversed by mutation to a frequent variant in tumours: m.16248T>C (1865/2) and m.16292T>C (1801/64). Finally, two mutations, m.195C>T (280/1574) and m.16519C>T, found in 3 samples (1115/752) occurred in positions that are variable in mitochondrial DNA, and both forms are common in the human population.

### Association of D-loop somatic mutations and patient clinicopathologic characteristics

Table 2 summarizes the presence of D-loop mutations according to clinicopathologic data, as evaluated by the two-tailed Fisher's exact test. No significant associations were found between the presence of mtDNA mutations and the histopathological tumour grading, presence of residual tumour, or mutation of *TP53*. No differences in tumour mutation frequencies were found between two groups of patients who were divided with respect to age below or above the median. No relation between mutational variability of this mtDNA region and response to chemotherapy was proven. A multivariate logistic regression model that contained haplogroup and mutation indicators also revealed no association of D-loop somatic mutations with any of the analyzed clinicopathologic parameters (data not shown).

### Association of mitochondrial gene expression with response to chemotherapy

**Figure 1:**
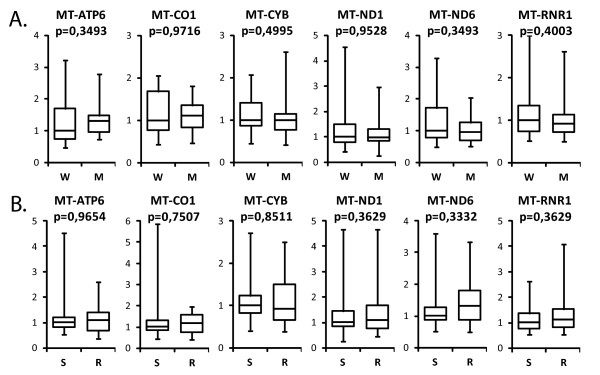
**Relative mRNA expression of selected genes in specimens from patients with ovarian carcinomas**. A. – samples divided according to mutational status of the mtDNA D-loop (*W *– wild type, n = 19; *M *– mutated, n = 25). B. – samples divided according to response to chemotherapy (*S *– sensitive, n = 32; *R *– resistant, n = 30). Data are presented as mRNA expression levels for selected genes normalized to the geometric mean of the RNA concentrations of three control genes, *glyceraldehyde-3-phosphate dehydrogenase*, *ubiquitin C*, and *β-actin*. The boxes show the upper and the lower quartiles; the median values are shown as a horizontal line in each box; the whiskers represent minimum and maximum of the data values. Data were analyzed with the Mann-Whitney *U *test.

Total RNA was isolated, and six transcripts that encoded polypeptides (*MT-ATP6, MT-CO1, MT-CYB, MT-ND1*, and *MT-ND6*) and ribosomal 12S RNA (*MT-RNR1*) were quantified by real-time RT-PCR. Because it was not possible to obtain sample pairs of ovarian carcinoma and corresponding normal tissue, gene expression was studied only in 62 tumour samples. Analysis of the results with the Mann-Whitney *U *test revealed that mutations in the D-loop did not influence the expression of mitochondrial genes (Fig. 1A). Furthermore, the expression of all analyzed mitochondrial genes was equal in responders and non-responders to the therapy (Fig. 1B). However, when the probability of sensitivity to chemotherapy was evaluated with a multivariate logistic regression model, both clinical parameters (age, FIGO stage, and grade) and the expression of *RNR1 *appeared to be predictors of platinum sensitivity (Table 3).

**Table 3:** Probability of sensitivity to chemotherapy in the group of 44 ovarian carcinomas as evaluated by the multivariate logistic regression model

	OR	95% CI for OR	*P*-value
Age			
≥ 53 vs. < 53	0.097	[0.02, 0.41]	0.002

Grade			
4 vs. 3, 2	0.15	[0.03, 0.76]	0.022

FIGO stage			
IIIC, IV vs. II, IIIB	0.099	[0.01, 0.64]	0.015

*RNR1 *expression			
> 1 vs. < 1	0.21	[0.05, 0.86]	0.031

Abbreviations: OR – odds ratio; CI – confidence interval.

## Discussion

In the present study, somatic mutations in the D-loop region of mtDNA were found in > 50% of ovarian cancer tissue samples, and most samples harbored a single mutation. These results are consistent with previous reports that described somatic D-loop mutations in 20–78% of human cancers [6,59,60]. However, the presence of these somatic mutations did not correlate with clinicopathologic parameters.

Views on the importance of mtDNA mutational variability in tumours vary from complete disbelief [61] through cautious acceptance of at least some data [62] to optimistic belief that mitochondrial studies in cancer patients might be employed to search for reliable disease markers [6]. Two major problems exist in several published studies. First, most of these studies analyzed relatively small groups. Second, some studies compared the sequences obtained from tumours with the Cambridge reference sequence instead of with corresponding germline controls, which yields obvious differences (e.g., Aikhionbare et al., 2007 [14]).

Brandon et al. [15] compiled an extensive list of mtDNA mutations identified in tumours and concluded that many of them are sequence polymorphisms found in databases. This was true for 85% of the mitochondrial control region mutations that were analyzed. Such mutations have occurred during human evolution and can be used for categorization according to mitochondrial haplogroups. We did not detect any significant association of a specific haplogroup with tumour occurrence. Thus far, convincing data have been found only by Booker et al. [63] for haplogroup U association with prostate and renal cancer. Although an association between haplogroups and predisposition to ovarian cancer could not be found, it should be noted that proving any association of mitochondrial haplogroups with a disease is extremely difficult. To obtain convincing evidence, very large patient and control groups are required, which are not available for most studies [64].

The unstable C-tract is located in the hypervariable segment II region of the D-loop of mtDNA, and length variations of this mononucleotide sequence are regarded as common polymorphisms. All sequence changes in the C-tract that were found in our study were insertions or deletions of one or two base pairs. Interestingly, although the unstable C-stretch is a hotspot for somatic mutation in ovarian cancers, as well as in other human cancers such as colorectal, lung, and liver [19,21,48,60,65,66], Sanchez-Cespedes et al. did not detect somatic insertions at the C-stretch sequence in ovarian and prostate cancers [66].

Most of the mutations identified in the present study are of the type described by Brandon et al. [15], i.e., changes in sites known to be polymorphic in the population. However, some of the mutations identified in the present study are very rare in the population databases (m.16193C>T, m.16218C>T, m.16391G>A, m.16465C>T, m.586G>A) or were not found in any database (m.565A>G, m.A567A>C, m.16034G>A). The scarcity of these mutations does not prove that they are cancer-specific, but they are at least uncommon. None of these mutations were found in ovarian cancers by Liu et al. [67], who found mutations in 20% (3 of 15) of paired tumour and normal tissue samples.

The functional significance of mtDNA mutations and their potential role in tumour development are still uncertain. In particular, it has not been established whether somatic mutations of mtDNA are involved in molecular mechanisms of tumour development or whether these mutations are merely associated with the tumourigenic process but lack a causative role.

## Conclusion

The present study showed for the first time that, in addition to clinical parameters, the expression of the mitochondrial gene *RNR1 *in tumour tissue might predict sensitivity of ovarian carcinomas to chemotherapy. However, the overall clinical relevance of mitochondrial molecular analyses in ovarian carcinomas seems to be rather limited. As pointed out by Salas et al. [61], the improper analysis and interpretation of mtDNA sequence data may lead to misinterpretations that, in turn, can be further duplicated from the published reports. In contrast to many previously published papers, our study indicates that the usefulness of estimation of the D-loop mtDNA sequence variability as a clinical cancer marker is questionable. Thus, the acceptance of any diagnostic mitochondrial molecular test for clinical practice requires additional large, well-designed validation studies.

## Competing interests

The authors declare that they have no competing interests.

## Authors' contributions

PB carried out the molecular studies and the sequence alignment, participated in the design of the study, statistical analysis and helped in drafting the manuscript. JK collected and characterized the material and participated in the design of statistical analysis. EB participated in the sequence alignment and drafting the manuscript. AR participated in molecular studies and the sequence alignment. JO participated in the design and coordination of the study, helped in the statistical analysis and drafted the manuscript. All authors read and approved the final manuscript.

## Pre-publication history

The pre-publication history for this paper can be accessed here:


